# System Delay in Breast Cancer Diagnosis in Gaza Strip, Palestine

**DOI:** 10.1155/2019/5690938

**Published:** 2019-12-11

**Authors:** Samira S. Abo Al-Shiekh, Yasser S. Alajerami, Bothyna B. Etewa, Aymen M. Elsous

**Affiliations:** ^1^Public Health Department, University of Al-Butana, Khurtom, Sudan; ^2^Department of Medical Imaging, Al-Azhar University, Gaza Strip, State of Palestine; ^3^Department of Obstetrics and Gynecological Nursing, University of Al-Butana, Somaliland, Sudan; ^4^Faculty of Health Professions, Israa University, Gaza Strip, State of Palestine; ^5^Unit of Planning an Policy Formulation, Ministry of Health, Gaza Strip, State of Palestine

## Abstract

**Background:**

Breast cancer is a major public health problem and the first leading cause of cancer deaths among females in Palestine. Early diagnosis of breast cancer contributes to reduction of morbidity and mortality rates. This study aimed to explore system-related factors affecting the timely diagnosis of breast cancer in the Gaza Strip.

**Method and Materials:**

A mixed method, sequential explanatory design was employed. A quantitative study was conducted first, and it was cross-sectional in nature, followed by a qualitative study. An interviewed questionnaire and an abstraction sheet were used to collect necessary quantitative data among 122 females diagnosed with breast cancer. A purposive sample of five medical specialists were selected for in-depth interview. Descriptive and inferential analyses were used to find differences between variables. Odds ratio and confidence interval at 95% were presented, and *P* < 0.05 was considered statistically significant.

**Results:**

Around 12.3% of women experienced diagnostic delay for 3 months and more, and 6.6% reported a delay in referral for more than 2 weeks. Regarding imaging delay, around 8.2% and 2.7% of women had reported a delay in performing mammography and ultrasound, respectively. Moreover, one-fourth reported delay in performing biopsy for more than 14 days, and 46.3% reported delay more than 14 days in getting histopathology report. In addition, 9% missed the follow-up after benign findings of the previous breast imaging and no national protocols are available for the diagnosis of breast cancer in the Gaza strip.

**Conclusion:**

There is a long appointment time for diagnostic tools especially in biopsy. The nonmalignant findings from mammography or ultrasound could affect diagnosis time. It is an urgent need to have a national protocol for diagnosis and management of breast cancer and to adopt screening, diagnostic, and follow-up programs under the supervision of the Ministry of Health.

## 1. Introduction

Breast cancer (BC) is a major public health problem especially among females in both developed and developing countries [1–4]. Early diagnosis of BC has a better prognosis, yielding a better survival rate [5, 6]. Globally in 2012, the incidence of BC was 1.67 million, and approximately half million deaths were reported [7]. In Palestine, 503 new BC cases were reported in the West Bank during 2017 which constituted around 17.2% of all registered cancer cases [8]. Moreover, 684 new cases of BC were registered in the Gaza strip during 2016 which represented 20.5% of all registered cancer cases [9].

The 5-year survival rate varies widely among countries, from 53% in South Africa to 89% in the United States of America [10]. The 5-year survival rate was reported to be 60–65% among Jordanian and Saudi Arabian women [11], 65.1% in the Gaza Strip [12], and 70% in Iran [13]. In return, it is better in developed countries: 82% in Europe [14] and 89% in the USA [15]. Unlike women in developed countries, women in less developed countries are diagnosed with BC when they are in an advanced stage because of poor of surveillance system and limited access to cancer diagnosis and treatment options [16–18].

Early diagnosis of BC is defined by the World Health Organization (WHO) as “early identification of patients with symptoms of BC without delay, patients with cancer should receive diagnostic examinations, pathological confirmation and staging procedures at a suitable diagnostic facility” [19]. Delay in diagnosis has bad implications and consequences. It is a delay for 3 months or more from first counseling visit until time of confirmation of diagnosis [20–22]. This study was conducted to explore system-related factors affecting the timely diagnosis of BC in the Gaza strip.

## 2. Methods and Materials

### 2.1. Study Design

This study was part of a master thesis submitted to School of Public Health, Al-Quds University in 2018 [23]. It was a mixed method, sequential explanatory design and involved both quantitative and qualitative study, in which the quantitative part was applied first followed by the qualitative study. This approach aimed to assess diagnosis delay and related factors and then to further understand and answer questions that rose from the findings of the quantitative study.

### 2.2. Study Setting

This study was carried out at Al-Rantisi and European Gaza hospitals (two main oncology governmental hospitals). Patients who have cancer receive their services and medical treatment including chemotherapy in these hospitals. They are located in Gaza city and Rafah, in the south of Gaza strip, respectively.

### 2.3. Study Sample and Sampling

Females newly diagnosed of BC were invited to participate in the study. Females should be diagnosed with BC no more than six months from the time of conducting this study and under medical treatment and follow up. They were 182 females in total, and we used the survey monkey online for calculation of sample size. It is available at https://www.surveymonkey.com/mp/sample-size-calculator/. Sample size was calculated with confidence interval at 95% and margin error at 5% and was estimated to be 124 females. No exclusion criteria were predetermined. Simple random sampling from a preprepared list was followed to select the participants.

Purposive sampling was also followed to select interviewees for the qualitative part of study. Five medical specialists (one radiologist, one oncologist, one histopathologist, one surgeon, and a general practitioner from primary health care center) were invited for interview.

### 2.4. Measurement and Study Period

Data were collected between January and December 2017. With regard to quantitative study, two tools for data collection were used: a semistructured interviewed questionnaire including open ended questions and an abstracting sheet. We searched for the relevant literature in PubMed and CINAHL database to build up the questionnaire. The questionnaire consisted of three parts: the first part was about sociodemographic characteristics and history of previous examinations. The second part was about signs and symptoms of BC. The third part contains questions about counseling of health care providers, number of consultations before diagnosis and referral, questions about diagnostic process and what had been done, delay time to seek health care, delay time to diagnosis, and appointments for imaging examinations. The questionnaire is attached and included in Appendix (supplementary [Supplementary-material supplementary-material-1]).

The abstraction sheet was developed and contained information on what had been done during diagnosis, date of examinations and reports, findings of diagnostic tests, biopsy date, results and dates of histopathology reports, and tumor stage (supplementary [Supplementary-material supplementary-material-1]).

In addition, a structured interview guide was developed to collect information on the diagnostic process, availability of protocols or guidelines for referring and diagnosis of BC, role of imaging tests in the diagnosis, patient delay in seeking health care and factors hinder seeking health care, and results of imaging tests and to what extent they are useful and effective in the diagnosis of BC.

### 2.5. Ethical Considerations

Ethical approval was obtained from the Palestinian Health Research Council (PHRC/HC/239/17). Permission was obtained from the Directorate General of Human Resources, Ministry of Health, to conduct the study in Al-Rantisi and European Gaza hospitals (158258/29/08/2017). Moreover, consent was taken from participated women and medical specialists. Study objectives were explained, and anonymity, rights, and confidentiality were ensured. Participated women were informed about their right to withdraw at any time they felt to do so. All gathered documents were kept and saved in a private closet.

### 2.6. Data Analysis

The statistical package for social sciences program (SPSS) version 23 was used for analysis. Data were checked for outliers and errors, and continuous variables were presented in forms of mean ± SD, while, categorical variables were presented in forms of frequency and percentage (*n*, %). Chi square (*X*^2^) was used to compare between two groups (delay and no delay of diagnosis), and *P* value was considered statistically significant at 0.05 level.

## 3. Results

### 3.1. Baseline Characteristics of Participants

One-hundred and twenty-two women participated. The mean (±SD) age was 51.2 ± 11.9 years (range 23–80 years), and more than half of them lied between 40 and 59 years old (56.5%). Quite half women were from Gaza city and the least from Rafah in the south (45.9% and 8.2%, respectively). Majority were married (68.9%) and unemployed (80.3%) and had health insurance (96.7%) (Table 1).

### 3.2. Forms of Delay

Fifteen women (12.3%) reported to have diagnostic delay of 35.8 ± 44.5 days (mean (±SD)), and eight (6.6%) had referral delay. However, a PHC doctor working at the mammography screening program excluded the occurrence of such delay. She said “*Doctors at PHC refer patients even though they did not have the actual signs and symptoms of BC, I do not expect referral delay to occur.*”

With regard to other types of delay, 8.2% (10/91) of women have reported delay in conducting mammography, 2.7% (3/111) reported delay in performing ultrasound (U/S), 25.9% (29/112) reported delay in performing biopsy, and 46.3% (56/121) reported delay in getting histopathology results (Table 2).

### 3.3. Reasons for Delay

The study findings showed that 9% of the participated women were not scheduled for follow up after diagnosis with benign tumors. Women explained this point as a barrier to early diagnosis as one woman said “*When I did mammography before 9 months, the doctor told me that I'm okay, however, he did not recommend me to come back in future for another test*.” Many specialists reported that failure in follow-up process is considered a diagnostic delay, as one of the oncologists said “*Absolutely, it is considered a delay in diagnosis, this woman should be at least programmed in a close follow up after benign findings in imaging or biopsy.*”

**Figure 1:**
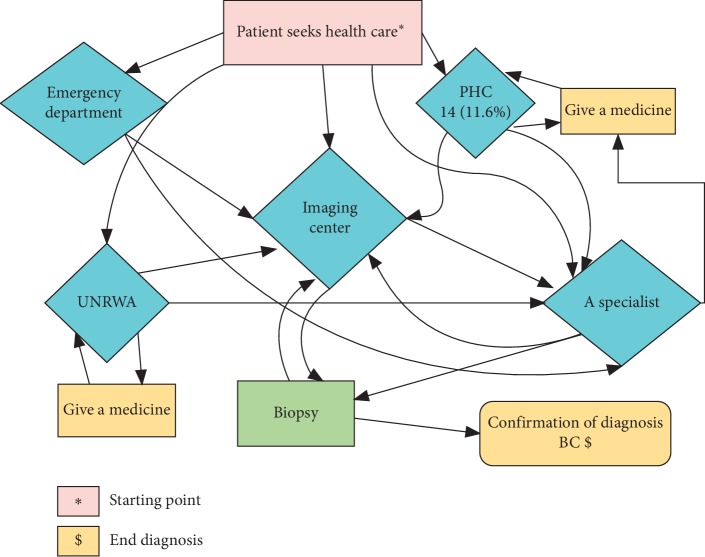
Referral process of suspected BC patients to imaging exams.

Insufficient education among physicians in the PHCs contributed to diagnosis delay. A general practitioner (GP) identified the problem in assessing BC in the PHC as she said “*GPs have insufficient education and training courses about BC assessment*.” Moreover, no obvious road map for referring suspected cases with BC and the process seems to be complex and fragmented (Figure 1).

Figures from Table 3 show that 40.2% of women were referred to medical imaging from first visit to a clinic, and 42.6% received two or three prereferral consultations to imaging diagnosis. In addition, 15.6% were counseled at least 4 times. Besides, around 26% underwent mammography and U/S at the same time followed by biopsy. In return, 25.4% did mammography, then U/S, and finally a biopsy, and 23.8% underwent first U/S followed by a biopsy.

### 3.4. Availability of National Protocols

Only 42% of patients were referred to diagnosis from the first consultation visit. About 42.6% of them necessitated two to three prereferral consultations, and 15% had prereferral consultations four times and more.

The assessment process of females with suspected BC is not consistent among physicians because there is no standard national protocol or guidelines to deal with these cases. A medical specialist said “*there is no clear guideline or protocol for what should be done. The choice is based on physician's experience and what he decides*.” A consultant radiologist specialized in breast imaging field said “*There are no written guidelines about BC diagnosis, the only documented guidelines stated in 2010 particularly for mammography screening program at PHC and it is not generalized for all health institutions*.” In return, physicians used various sources and some relied on the European guidelines, while others followed the American guidelines in the diagnosis and follow up of suspected cases with BC. One specialist stated “*There is no generalized protocol for all the institutions, but we follow European guidelines and some follow American guidelines in the diagnosis process and follow up also*.”

### 3.5. Independent Factors Associated with Diagnosis Delay

Univariate analysis of independent factors revealed age less than 40 years and nonmalignant findings in mammography are high potentials for diagnosis delay (OR: 2.7, CI: 95% 1.07–6.9) and (OR: 8.3, CI: 95% 2.3–30), respectively, (*P* < 0.05). Women who had wrong report of nonmalignant findings in mammography had diagnostic delay eight times more. In addition, women who reported nonmalignant findings from U/S test were almost four times more potential for diagnostic delay (OR: 3.73; CI: 1.4–9.5, *P* < 0.018). Other independent variables like place of residence, income, education level, and the presence of family history of BC were not statistically significant (*P* < 0.05) (Table 4).

## 4. Discussion

Women in Gaza experienced BC mostly at age 51, which is 10 years less than those in developed countries [24, 25]. Usually, access to medical care is limited and the diagnosis is made late when the disease is in advance stages, and therefore mortality from BC increases [26]. The study showed that 12% of women experienced diagnostic delay three months and more. In comparison, our rate is considered very low and far away from the previous findings from Malaysia (72.6%) [27] and Thailand (42%) [21].

The study showed that there are differences in the way of referral to imaging methods. Findings of our study revealed that referral to imaging exams did not follow international guidelines, and there is a need to unify it among the health care institutions. The best practice guidelines recommend health care workers to take the history first and perform clinical breast examination before generating further images. According to age, if a woman is 40 years old or more, she should do mammography first and then U/S in the initial assessment of breast disease. In contrast, woman less than 40 years old and is suspicious to have breast malignancy should start with U/S and then a mammography [28]. The World Health Organization reported lack of skill to perform clinical examination as a contributing factor to misdiagnosis and/or delay in diagnosis. The health care provider must have an appropriate index of suspicion, clinical skills, and resources to make an accurate diagnosis [19].

The study showed that half of the participated women were referred for imaging after many consultations which is also determined as a delay in diagnosis. Inconsistent to this, Lyratzopoulos et al. [29] found that patients with breast cancer had three or more prereferral consultations (3% and 5%, respectively) before performing further examinations. The study revealed that some types of delay occurred during the conduction of breast exam. Such delays may occur as a result of exam appointment or because of nonmalignant findings of a previous imaging test. The proposed protocol for benign lesions in a mammography is the clinical follow-up or repetition of the exams in other cases [30, 31].

Women's age was found to be a main factor affecting BC diagnosis. The younger the women are, the more delay in diagnosis is reported. Possible explanation is that breast density decreases with age and therefore mammography became more sensitive and thus physicians give more attention and priorities to adults and old-aged women than youngsters. This result is consistent with findings of Barber and his colleagues [32–34] and Ermiah et al. [35]. However, Dianatinasab and her colleagues [36] showed that age is not an independent factor attributed to the late stage.

Likewise, nonmalignant findings, either in mammography or U/S, could affect the early diagnosis of BC, and three main scenarios are expected neglection, treatment as a disease rather than a cancer, or scheduled in a close follow-up program. All these management and follow-up processes may delay the time of actual diagnosis. Therefore, nonmalignant findings either in mammography or U/S increase the delay time to diagnosis [27, 34].

## 5. Conclusion and Recommendations

Diagnosis of breast cancer is affected by interrelated factors including referral, inactive standard protocol, women's age, long appointment time for diagnostic tools especially biopsy, and nonmalignant findings of mammography or ultrasound. There is a need to unify guidelines for screening, diagnosis, and follow-up procedures in order to assure provision of timely and accurate care for breast cancer. Also, there is a need to minimize appointment time for imaging diagnostic tools and biopsy.

## Figures and Tables

**Table 1:** Demographic characteristics of study participants (*N* = 122).

Variable		*N* (%)
Age groups	<40	19 (15.6)
	40–49	36 (29.5)
	50–59	33 (27)
	60–69	22 (18)
	70 and more	12 (9.9)

Place of residence	North Gaza	17 (13.9)
	Gaza	56 (45.9)
	Middle zone	24 (19.7)
	Khan Younis	15 (12.3)
	Rafah	10 (8.2)

Marital status	Single	12 (9.8)
	Married	84 (68.9)
	Others	26 (21.3)

Level of education	Primary and less (0–6 classes)	29 (23.8)
	Preparatory or secondary (7–12 classes)	62 (50.8)
	University and above	31 (25.4)

Employment status	Yes	24 (19.7)
	No	98 (80.3)

Income (NIS^*∗*^)	>1000 NIS	59 (51.3)
	1000–2290 NIS	32 (27.8)
	≥2290 NIS	24 (20.9)

Presence of health insurance	Yes	118 (96.7)
	No	4 (3.3)

^*∗*^NIS: new Israeli shekel.

**Table 2:** Distribution of cases by potential delay categories.

Type of delay	Category	Frequency (%)
Diagnostic delay, *n* = 122	≥3 months	15 (12.3)
Referral delay, *n* = 122	<14 days	8 (6.6)
Mammography delay, *n* = 91	<7 days	10 (8.2)
U/S delay, *n* = 111	<7 days	3 (2.7)
Biopsy delay, *n* = 112	<14 days	29 (25.9)
Histopathology delay, *n* = 121	<14 days	56 (46.3)

**Table 3:** Referral of suspected BC patients to diagnosis (*N* = 122).

Variable		*N* (%)
Number of counseling times before referral to imaging service	1 time	49 (40.2)
	2-3 times	52 (42.6)
	≥4 times	19 (15.6)
	Did not counsel	2 (1.6)

Utilized diagnostic modalities	Mammography + U/S + biopsy	79 (64.8)
	U/S + biopsy	30 (24.6)
	Mammography + biopsy	12 (9.8)
	Only biopsy	1 (0.8)

Ranking for utilized imaging modalities	Combined mammography and U/S-biopsy	32 (26.3)
	Mammography-U/S-Biopsy	31 (25.4)
	U/S-biopsy	29 (23.8)
	U/S-mammography-biopsy	14 (11.5)
	Mammography-biopsy	10 (8.2)
	Biopsy-U/S	2 (1.6)
	U/S-biopsy-mammography	2 (1.6)
	Biopsy	1 (0.8)
	U/S-biopsy-combined mammography and U/S	1 (0.8)

**Table 4:** Relationship between diagnostic delay and demographic variables.

Variable	Categories	Diagnostic delay		OR with CI	*P* value
		Delayers ≥3 months	Nondelayers <3 months		
Age	<40	18 (16.8)	76 (40.0)	2.7 (1.07–6.9)	0.045^*∗*^
	≥40	9 (60.0)	89 (83.2)		

Place of residence	North Gaza	2 (1.6)	15 (12.3)	0.47 (0.02–10.27)	0.64
	Gaza	7 (5.7)	49 (40.2)	1.42 (0.12–16.57)	0.78
	Middle zone	4 (3.3)	20 (16.4)	2.18 (0.15–31.98)	0.57
	Khan Younis	1 (0.8)	14 (11.5)	0.4 (0.02–8.54)	0.56
	Rafah	1 (0. 8)	9 (7.4)	Ref.	

Income	<1000 NIS	10 (83.4)	49 (47.6)	12.17 (1.23–120.43)	0.03
	1000–2290 NIS	1 (8.3)	31 (30.1)	0.79 (0.04–14.11)	0.87
	≥2290 NIS	1 (8.3)	23 (22.3)	Ref.	

Level of education	Primary and less (0–6 classes)	0 (0.0)	29 (23.8)	Ref.	
	Preparatory or secondary (7–12 classes)	9 (7.4)	53 (43.4)	1.1 (1–1.2)	0.053
	University and above	6 (4.9)	25 (20.5)	1.2 (1.04–1.47)	0.015

A family history of BC	No	9 (60.0)	72 (67.3)	1.3 (0.45–4.1)	0.39
	Yes	6 (40.0)	35 (32.7)		

Mammography findings	Malignant findings	6 (6.8)	11 (12.5)	8.3 (2.3–30)	0.001^*∗*^
	Nonmalignant findings	3 (3.4)	68 (77.3)		

U/S Findings	Malignant findings	9 (64.3)	85 (90.4)	3.73 (1.4–9.5)	0.018^*∗*^
	Nonmalignant findings	5 (35.7)	9 (9.6)		

^*∗*^Statistically significant; ^∋^Fisher's exact test; NIS: new Israeli shekel.

## Data Availability

The data used to support the findings of this study are available from the corresponding author upon request.

## Supplementary Materials

Supplementary Materials The file is about the interviewed questionnaires with the patients. The other one is the in-depth interviews questions with the specialists. Supplementary file 1: the questionnaire sheet; supplementary file 2: the abstraction sheet.
