# Supplementary material for: System Delay in Breast Cancer Diagnosis in Gaza Strip, Palestine
Source: J Oncol. 2019 Dec 11;2019:5690938. doi: 10.1155/2019/5690938 (PMC6927013; doi:10.1155/2019/5690938)
Supplement: Supplementary Materials — The file is about the interviewed questionnaires with the patients. The other one is the in-depth interviews questions with the specialists. Supplementary file 1: the questionnaire sheet; supplementary file 2: the abstraction sheet. [file 5690938.f1.zip › 5690938.f1/abstraction sheet.docx]

**Abstraction sheet**

| **Item** | | **Mammography** | **U/S** | **MRI** |
| --- | --- | --- | --- | --- |
|  | Request date(day/month/year) |  |  |  |
|  | Examination date(day/month/year) |  |  |  |
|  | Report date(day/month/year) |  |  |  |
|  | Report conclusion  1.Normal  2.Benign findings  3.Dense breast for other investigation  4.Suspected Malignancy |  |  |  |
|  | BI-RADS classification |  |  |  |
|  | Next step |  |  |  |
|  | Is the examination requested for the patient in need? | 󠄞 Yes  󠄞No | 󠄞 Yes  󠄞No | 󠄞 Yes  󠄞No |
|  | If the exam is not needed, explain why? |  | | |
|  | Biopsy Date | / / (day/month/year) | | |
|  | Histopathology Report Date | / / (day/month/year) | | |
|  | Biopsy procedure | 󠄞 FNA  󠄞 True cut  󠄞 Both (FNA+ True cut)  󠄞Excision B. | | |
|  | How many biopsies were needed to confirm diagnosis? |  | | |
|  | Cancer Type | ____________ | | |
|  | Cancer stage | 󠄞 I  󠄞 II  󠄞 III  󠄞 IV | | |
