# Supplementary material for: System Delay in Breast Cancer Diagnosis in Gaza Strip, Palestine
Source: J Oncol. 2019 Dec 11;2019:5690938. doi: 10.1155/2019/5690938 (PMC6927013; doi:10.1155/2019/5690938)
Supplement: Supplementary Materials — The file is about the interviewed questionnaires with the patients. The other one is the in-depth interviews questions with the specialists. Supplementary file 1: the questionnaire sheet; supplementary file 2: the abstraction sheet. [file 5690938.f1.zip › 5690938.f1/questionnaire.docx]

**Interviewed questionnaire**

A. Serial number:

B. Oncology center: Al-Rantesi H. Gaza European H.

Patient name:

ID number:

Contact number:

Date of the interview: / / (day/month/year)

| 1. **First: Interviewed Questionnaire** | | | | | | | | | | | | | | | |
| --- | --- | --- | --- | --- | --- | --- | --- | --- | --- | --- | --- | --- | --- | --- | --- |
| 1. **Personal Data** | | | | | | | | | | | | | | | |
| 1.1 | | Age | | | | 󠄞years | | | | | | | | | |
| 1.2 | | Permanent place of residence | | | | 󠄞 North Gaza  󠄞Gaza  󠄞Middle Zone  󠄞Khanyounis  󠄞Rafah | | | | | | | | | |
| 1.3 | | Marital Status | | | | 󠄞Single  󠄞Married  󠄞Divorced  󠄞Widow | | | | | | | | | |
| 1.4 | | Level of education | | | | 󠄞Illiterate  󠄞Primary school (1-6 classes)  󠄞Preparatory school (7-9 classes)  󠄞Secondary education (10-12 classes)  󠄞University education | | | | | | | | | |
| 1.5 | | Number of children | | | | **󠄞** | | | | | | | | | |
| 1.6 | | Are you working? | | | | 󠄞 Yes  󠄞 No  If yes, specify your job: ________________ | | | | | | | | | |
| 1.7 | | Monthly average household income | | | | 󠄞 | | | | | | | | | |
| 1.8 | | Do you have a health insurance? | | | | 󠄞 Yes  󠄞 No  **If yes, what is its type?**  󠄞compulsory  󠄞israeli workers  󠄞Voluntary  󠄞MOSA  󠄞Other, Specify________________ | | | | | | | | | |
| 1. **Medical history** | | | | | | | | | | | | | | | |
| 2.1 | Do you have a family history of breast cancer? | | | | | 󠄞No  󠄞Yes  If yes, what is the relation?  **More than one option is possible**  󠄞Mother  󠄞Sister  󠄞Daughter  󠄞Aunt  󠄞Grandmother  󠄞Second degree relative | | | | | | | | | |
| 2.2 | In which side the problem was? | | | | | 󠄞RT breast  󠄞 LT breast  󠄞 Both | | | | | | | | | |
| 2.3 | What were the Signs and Symptoms at the time of diagnosis? | | | | | Symptom | | | | | | | | Yes | No |
|  |  |  |  |  |  | 󠄞Breast mass  󠄞Mass under axilla  󠄞Pain  󠄞Tingling  󠄞Nipple discharge  󠄞Retracted nipple  󠄞Two breast are not equal  in size or shape  󠄞Readiness  󠄞asymptomatic | | | | | | | |  |  |
|  |  |  |  |  |  | Others, Specify: ____________ | | | | | | | | | |
| 2.4 | What was the time interval between the appearance of signs and symptoms and seeking health care services? | | | | | 󠄞____________ | | | | | | | | | |
| 2.5 | Answer with yes or no about barriers that may face you during the diagnostic process: | | | | | 󠄞 symptom was not serious  󠄞Shy demonstrating  symptoms to health care professionals  󠄞 Lack of pain  󠄞 No chief complaint  󠄞 Stigma  󠄞 Feared of results  󠄞 diagnostic facility was too far  󠄞 Complexity of referral system  󠄞 Don’t know where to go  󠄞 Service was not available  󠄞 Fear of husband abandonment  󠄞 I went to traditional healers  󠄞 Cost of the exams  󠄞Transportation costs  󠄞 Didn’t trust of health care system  󠄞Lack of female health care providers  󠄞 My husband prevented me  󠄞 Fear of exposure to radiation  󠄞 Fear of pain related to the exams  󠄞I don’t able to organize my time  󠄞 A previous doctor visit who did not take care of me  󠄞 A previous examination and the results were free  For other reasons  Specify, ________________ | | | | | | | | Yes | No |
| 2.6 | How long did it take for completion the diagnosis?  (from seeking health care to be diagnose of BC) | | | | | 󠄞 | | | | | | | | | |
| 2.7 | Who encourage you to seek services? | | | | | 󠄞My husband  󠄞 Family  󠄞A screening program at a health facility  󠄞 no one (Self) | | | | | | | | | |
| 2.8 | Referral doctor specialty for the first diagnostic modality | | | | | 󠄞PHC GP  󠄞Surgeon  󠄞 Oncologist  󠄞Gynecologist  󠄞 Radiologist  󠄞UNRWA  󠄞Screening program at NGOs  Others, Specify _____________ | | | | | | | | | |
| 2.9 | How many times did you counsel health care providers before starting diagnostic process? | | | | | 󠄞 times | | | | | | | | | |
| 2.10 | Which exam(s) did you do during the diagnostic process?  **(more than one answer is possible)** | | | | | 󠄞mammography  󠄞 U/S  󠄞MRI  󠄞Biopsy | | | | | | | | | |
| 2.11 | Please, order the exams that have been performed to you during the diagnostic process,,  (1, 2, 3, 4) | | | | | 󠄞Mammography  󠄞 U/S  󠄞 combined mammography and U/S  󠄞MRI  󠄞Biopsy | | | | | | | | | |
| 2.12 | Have you ever been examined before? | | | | | 󠄞No (If answer is no, move to question number (3.1)  󠄞Yes  󠄞No  If yeas (for what reason?)  󠄞Screening  󠄞Diagnostic | | | | | | | | | |
| 2.13 | What was the exam (Exams) done? | | | | | 󠄞Mammography  󠄞U/S  󠄞Biopsy | | | | | | | | | |
| 2.14 | When did you have a previous examination? | | | | | 󠄞 | | | | | | | | | |
| 2.15 | What was the result? | | | | | 󠄞Normal  󠄞Benign findings  󠄞Calcifications | | | | | | | | | |
| 2.16 | Where did you perform the previous examination (s)? | | | | | 󠄞Governmental hospital  󠄞 NGOs  󠄞 Private sector | | | | | | | | | |
| 1. **Accessibility and Affordability data** | | | | | | | | | | | | | | | |
| 3.1 | How many times did you visit the hospital or clinic to complete the diagnostic process? | | | | | 󠄞 times | | | | | | | | | |
| 3.2 | Did you receive a financial support from anyone to complete the diagnostic process? | | | | | 󠄞 No  󠄞Yes  Specify whom? | | | | | | | | | |
| 3.3 | Did you change the facility during the diagnostic process? | | | | | 󠄞 No  󠄞Yes  If yes, for what diagnostic exam?  󠄞Mammography  󠄞U/S  󠄞MRI  󠄞Biopsy | | | | | | | | | |
| 3.4 | What was the reason for changing the diagnostic facility? | | | | | --------------------------------------------- | | | | | | | | | |
| 1. **General Questions about the diagnostic modalities** | | | | | | | | | | | | | | | |
| 4.1 | During the diagnostic process, Where did you do the exams? | | | | | | | | | | | | | | |
| **Mammography** | | | **U/S** | | | | | **MRI** | | | **Biopsy** | | | | |
| 󠄞Yes 󠄞No | | | 󠄞Yes 󠄞No | | | | | 󠄞Yes 󠄞No | | | 󠄞Yes 󠄞No | | | | |
| 󠄞Governmental h.  󠄞NGOs  󠄞NGOs with free breast exam  󠄞Private sector | | | 󠄞Governmental h.  󠄞NGOs  󠄞NGOs with free breast exam  󠄞Private sector | | | | | 󠄞Governmental h.  󠄞NGOs  󠄞NGOs with free breast exam  󠄞Private sector | | | 󠄞Governmental h.  󠄞NGOs  󠄞NGOs with free breast exam  󠄞Private sector | | | | |
| 4.2 | Why did you choose this sector to perform the exam? | | | | | | | | | | | | | | |
| **Mammography** | | | | **U/S** | | | **MRI** | | | | **Biopsy** | | | | |
| 1-more affordable  2- No long appointment  3- Trust  4- More quality  5- doctor advise  6- health insurance  7- One of my family members advice  8. Availability of the service  9. It is close to my Place of residence  10.Society referral  11. UNRWA referral | | | | 1-more affordable  2- No long appointment  3- Trust  4- More quality  5- doctor advise  6- health insurance  7- One of my family members advice  8. Availability of the service  9. It is close to my Place of residence  10.Society referral  11. UNRWA referral | | | 1-more affordable  2- No long appointment  3- Trust  4- More quality  5- doctor advise  6- health insurance  7- One of my family members advice  8. Availability of the service  9. It is close to my Place of residence  10.Society referral  11. UNRWA referral | | | | 1-more affordable  2- No long appointment  3- Trust  4- More quality  5- doctor advise  6- health insurance  7- One of my family members advice  8. Availability of the service  9. It is close to my Place of residence  10.Society referral  11.UNRWA referral | | | | |
| 4.3 | How many days did you wait to perform the exams? ( Appointment) | | | | | | | | | | | | | | |
| Mammography | | | U/S | | | | | MRI | | | | Biopsy | | | |
|  | | |  | | | | |  | | | |  | | | |
| 4.4 | How much did you pay for the diagnostic exams? | | | | | | | | | | | | | | |
| Mammography | | | U/S | | | | | MRI | | | | Biopsy | | | |
| ( ) NIS | | | ( ) NIS | | | | | ( ) NIS | | | | ( ) NIS | | | |
| 4.5 | How much did you pay to transportation for diagnostic facilities? (Including all visits for all exams) | | | | | | | | | | | ( ) NIS | | | |
| 4.6 | In general what is your satisfaction about the quality level of the service provided? | | | | **Mammography**  *1. less than accepted 2.accepted*  *3. good*  *4.Excellent* | | | | **U/S**  *1. less than accepted 2.accepted*  *3. good*  *4.Excellent* | **MRI**  *1. less than accepted 2.accepted*  *3. good*  *4.Excellent* | | | **Biopsy**  *1. less than accepted 2.accepted*  *3. good*  *4.Excellent* | | |
| 4.7 | Will your recommend this service to one of your friends or family if it is needed? | | | | 󠄞Yes  󠄞No | | | | 󠄞Yes  󠄞No | 󠄞Yes  󠄞No | | | 󠄞Yes  󠄞No | | |

4.8 What are perceived barrierthat could hinder your diagnostic process?

------------------------------------------------------------------------------------------

4.9 Did you have any suggestion that could enhance the quality of the services provided?

--------------------------------------------- ---------------------------------------------

| **Domain** | **No.** | **Statement** | **Mammography** | **U/S** | **MRI** | **Biopsy** |
| --- | --- | --- | --- | --- | --- | --- |
|  |  |  | *1.Strongly*  *disagree*  *2.Disagree*  *3.Uncertain*  *4.agree*  *5.strongly*  *agree* | *1.Strongly disagree*  *2.Disagree*  *3.Uncertain*  *4.agree*  *5.strongly*  *agree* | *1.Strongly disagree*  *2.Disagree*  *3.Uncertain*  *4.agree*  *5.strongly agree* | *1.Strongly disagree*  *2.Disagree*  *3.Uncertain*  *4.agree*  *5.strongly*  *agree* |
| **Physical Accessibility and affordability** | 1. | It was easy to reach to the center |  |  |  |  |
|  | 2. | The distance between your place of residence and the facility was suitable |  |  |  |  |
|  | 3. | Transportation was available to the diagnostic facility |  |  |  |  |
|  | 4. | In general, the performance of health care providers was good |  |  |  |  |
|  | 5. | The cost of the exam was reasonable for you |  |  |  |  |
|  | 6. | The transportation cost to reach the hospital was suitable |  |  |  |  |
| **Appointment and waiting time** | 7. | The referral system to diagnostic facility was within appropriate time |  |  |  |  |
|  | 8. | The appointment to conduct the exam was suitable for you |  |  |  |  |
|  | 9. | Health care providers committed with the appointments |  |  |  |  |
|  | 10 | Waiting time to get the service was appropriate |  |  |  |  |
|  | 11. | The results of medical imaging exam were received at an appropriate time |  |  |  |  |
| **Communication and Patients' rights** | 12. | Health care provider introduced him/ her- self before conducting the exam |  |  |  |  |
|  | 13. | Medical imaging procedure was explained by the health care provider |  |  |  |  |
|  | 14. | Health care provider answered your questions carefully |  |  |  |  |
|  | 15. | Clean gown and coverlet were available |  |  |  |  |
|  | 16. | Privacy was valued during imaging procedure |  |  |  |  |
|  | 17. | There were female health care providers |  |  |  |  |
|  | 18. | You were given enough time to explain your condition |  |  |  |  |
|  | 19. | No discrimination between patients |  |  |  |  |
|  | 20. | Feasible contact with the diagnostic facility |  |  |  |  |
